# Sea Urchin Injury Complicated by Lime Phototoxic Reaction

**DOI:** 10.4269/ajtmh.23-0509

**Published:** 2023-11-13

**Authors:** Yasmin Alfallouji, Stephen L. Walker

**Affiliations:** ^1^ Department of Dermatology, University College London Hospitals NHS Foundation Trust, London, United Kingdom;; ^2^ Department of Infectious and Tropical Diseases, Hospital for Tropical Diseases, University College London Hospitals NHS Foundation Trust, London, United Kingdom;; ^3^ Faculty of Infectious and Tropical Diseases, London School of Hygiene and Tropical Medicine, London, United Kingdom

**Figure 1.**
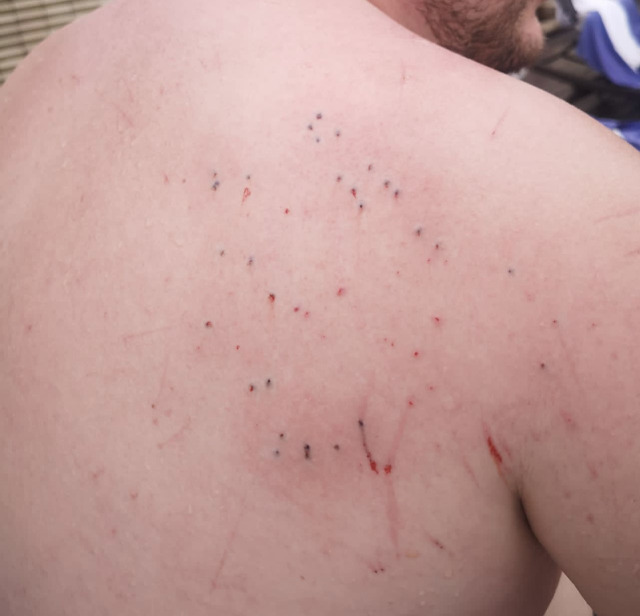
Immediately following sea urchin injury highlighting sea urchin spines right upper back.

**Figure 2.**
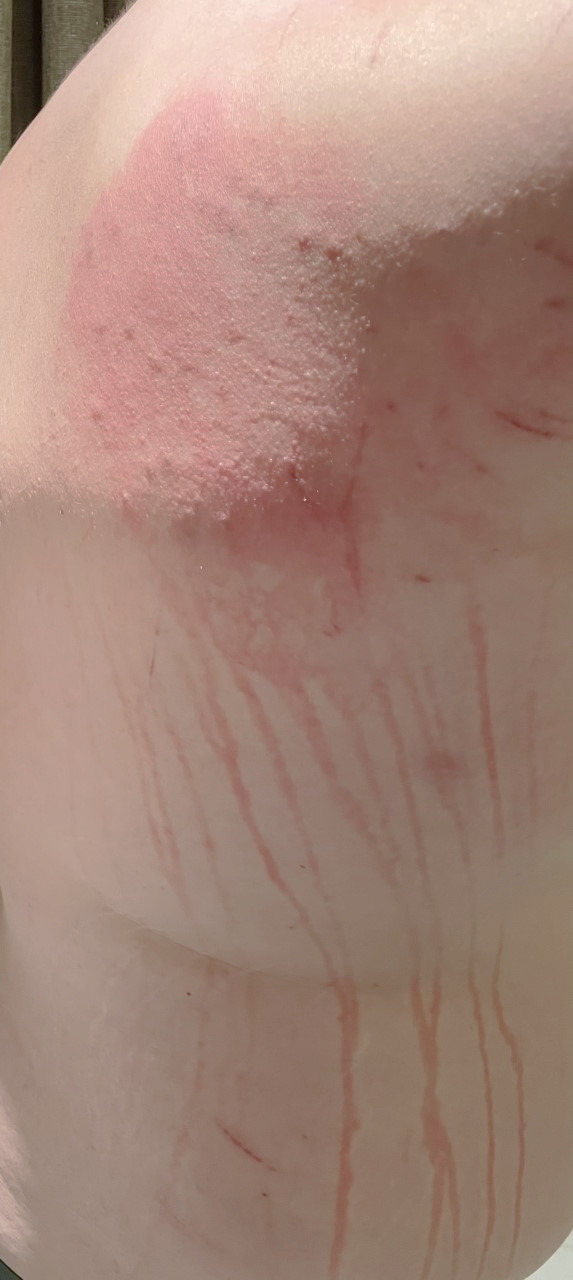
Two days after sea urchin injury- depicting erythematous patch and streaking at site of lime juice contact.

**Figure 3.**
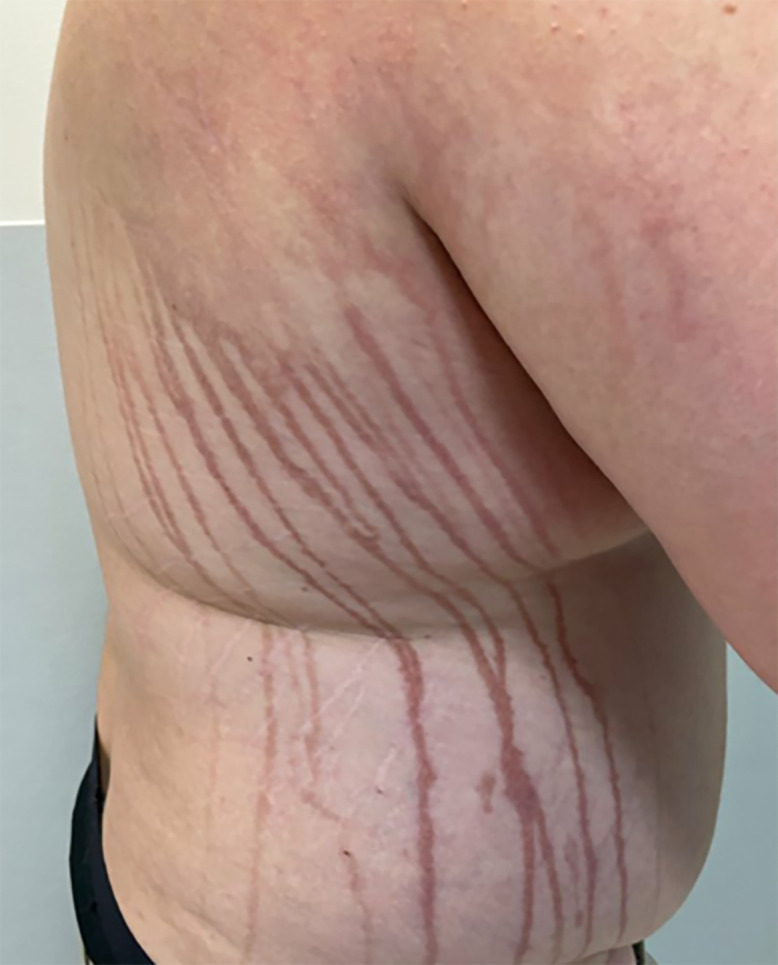
Four weeks after exposure depicting post-inflammatory changes.

A 29-year-old man sustained a sea urchin injury (SUI) while swimming in the sea in Thailand. The hotel doctor promptly removed the spines (Figure 1); however, over the next 48 hours the site of the SUI became inflamed, and discrete, linear, erythematous macules extended from the area of inflammation (Figure 2). Four weeks later, concerned by the persistent linear streaks resulting from the SUI (Figure 3), he was referred to our emergency dermatology service.

The skin affected by the SUI was normal, but there were hyperpigmented, linear macules consistent with postinflammatory hyperpigmentation. There was no pain or pruritus. On direct questioning, he recalled that the hotel doctor had applied lime juice to the area to aid in removal of remnant sea urchin spines. The procedures had been conducted outside, exposing the lime juice to sunlight while in contact with his skin, causing these unusual changes.

Phytophotodermatitis is a non–immune-mediated cutaneous eruption that develops after exposure to ultraviolet A (UVA) radiation after contact with phototoxic agents found in certain plants.^1^ Furocoumarins are one example of such an agent; they are naturally occurring compounds found in many plants. They are found in large quantities in citrus species, including bergamot oranges and limes, and sensitize epithelial DNA to UVA radiation.^1^ On the skin, furocoumarins are excited into a reactive state when exposed to UVA light. These photoactivated compounds bind to nucleic acids, leading to inhibition of DNA synthesis; a second mechanism that causes direct cell membrane damage also occurs. The result is cell death and epidermal injury.^2^

Diagnosis of phytophotodermatitis is clinical based on characteristic skin eruption and detailed history of potential exposures. It is characterized by the appearance of erythematous macules, or patches, that are often linear or irregularly shaped and well demarcated, pertaining to the area of contact. Blistering may occur, and hyperpigmentation is common.^3^ Phytophotodermatitis is self-limiting, but initial treatment includes topical corticosteroids.^3^

The SUI was appropriately treated by removal of the spines,^4^ but the application of lime juice caused the skin changes seen in our patient. In nonmedical settings such as beach resorts, use of lime juice or other acidic substances to attempt to dissolve the spines is believed to be an effective method. Removal of the spines is crucial to prevent development of chronic granulomatous reactions,^4^ but use of acidic substances such as lime juice should be avoided. Use of nonphotosensitizing antiseptic agents may be more appropriate. This case highlights the importance of obtaining a detailed history in returning travelers with skin disorders.
